# Identification and Characterization of (3*Z*):(2*E*)-Hexenal Isomerases from Cucumber

**DOI:** 10.3389/fpls.2017.01342

**Published:** 2017-08-02

**Authors:** Eleni A. Spyropoulou, Henk L. Dekker, Luuk Steemers, Jan H. van Maarseveen, Chris G. de Koster, Michel A. Haring, Robert C. Schuurink, Silke Allmann

**Affiliations:** ^1^Department of Plant Physiology, Swammerdam Institute for Life Sciences, University of Amsterdam Amsterdam, Netherlands; ^2^Department of Mass Spectrometry of Biomacromolecules, Swammerdam Institute for Life Sciences, University of Amsterdam Amsterdam, Netherlands; ^3^Department of Synthetic Organic Chemistry, Van ’t Hoff Institute for Molecular Sciences, University of Amsterdam Amsterdam, Netherlands

**Keywords:** green leaf volatiles, *E*-2-hexenal, (*E*, Z)-2, 6-nonadienal, cucumber, hexenal isomerase, purification

## Abstract

*E*-2-hexenal is a volatile compound that is commonly emitted by wounded or stressed plants. It belongs to the group of so-called green leaf volatiles (GLVs), which play an important role in transferring information to plants and insects. While most biosynthetic enzymes upstream of *E*-2-hexenal have been studied extensively, much less is known about the enzyme responsible for the conversion from *Z*-3- to *E*-2-hexenal. In this study we have identified two (3*Z*):(2*E*)-hexenal isomerases (HIs) from cucumber fruits by classical biochemical fractionation techniques and we were able to confirm their activity by heterologous expression. Recombinant protein of the HIs did not only convert the leaf aldehyde *Z*-3-hexenal to *E*-2-hexenal, but also (*Z,Z*)-3,6-nonadienal to (*E,Z*)-2,6-nonadienal, these last two representing major flavor volatiles of cucumber fruits. Transient expression of the cucumber HIs in *Nicotiana benthamiana* leaves drastically changed the GLV bouquet of damaged plants from a *Z*-3- to an *E*-2-enriched GLV profile. Furthermore, transcriptional analysis revealed that the two HIs showed distinct expression patterns. While *HI-1* was specifically expressed in the flesh of cucumber fruits *HI-2* was expressed in leaves as well. Interestingly, wounding of cucumber leaves caused only a slight increase in *HI-2* transcript levels. These results demonstrate that cucumber HIs are responsible for the rearrangement of *Z*-3-aldehydes in both leaves and fruits. Future research will reveal the physiological importance of an increased conversion to *E*-2-aldehydes for plants and insects.

## Introduction

When plants are stressed they respond, amongst others, with the release of volatiles (Baldwin, 2010; Maffei et al., 2011; Ameye et al., 2017). This increase in plant volatiles enables them to interact with its biotic environment (Heil, 2014; Pickett and Khan, 2016). GLVs are produced by almost every plant and represent an important group of plant volatiles. While unstressed plants emit only small amounts, the release of GLVs can rapidly increase upon cell damage and other types of (a)biotic stresses (Scala et al., 2013a; Matsui and Koeduka, 2016). GLVs have been shown to play an important role in the activation (Bate and Rothstein, 1998; Farag and Paré, 2002; Engelberth et al., 2013) or priming (Engelberth et al., 2004; Kost and Heil, 2006; Ameye et al., 2015; Li et al., 2016) of defense responses in plants. They can influence the performance of pathogens (Shiojiri et al., 2006; Kishimoto et al., 2008; Scala et al., 2013b) and herbivores (Vancanneyt et al., 2001; Halitschke et al., 2004) and serve as infochemicals for beneficial (Turlings et al., 1991; Brodmann et al., 2008; Allmann and Baldwin, 2010) and detrimental (Halitschke et al., 2008; Li et al., 2012) insects.

Green leaf volatiles are formed via the oxylipin pathway from C18-polyunsaturated fatty acids α-linolenic and linoleic acid (Hatanaka et al., 1987; Scala et al., 2013a; ul Hassan et al., 2015). Molecular oxygen is incorporated into free or membrane-bound fatty acids by 13-lipoxygenases and the resulting 13-hydroperoxides are cleaved through the action of HPL in a C12- (12-oxo-(Z)-9-dodecenoic acid) and a C6- (*n*-hexanal or Z-3-hexenal) compound (Nakashima et al., 2013; Mwenda and Matsui, 2014). *Z*-3-hexenal, which is formed from α-linolenic acid, can be further converted to its alcohol and esters (Bate et al., 1998; D’Auria et al., 2007; Matsui et al., 2012), but it can also rearrange either spontaneously or through catalysis by a (3*Z*):(2*E*)-hexenal isomerase (HI) to *E*-2-hexenal (Allmann and Baldwin, 2010; Kunishima et al., 2016). Such isomerase activity has recently been identified in the oral secretions of the lepidopteran caterpillar *Manduca sexta* (Allmann and Baldwin, 2010): when feeding from their host plants, e.g., *Nicotiana attenuata* or *Datura wrightii*, their oral secretions are introduced into the leaf wounds and *Z*-3-hexenal which is quickly formed from damaged plant material is readily converted to *E*-2-hexenal and the corresponding *E*-2-derivatives (Allmann and Baldwin, 2010; Allmann et al., 2013). This OS-induced change in the *Z*-3-/*E*-2-ratio increased the foraging efficiency of the generalist predator *Geocoris* spp. (Allmann and Baldwin, 2010) and decreased the oviposition rate of female adult moths in nature (Allmann et al., 2013).

Not only insects, but also plants are able to catalyze the conversion from *Z*-3- to *E*-2-hexenal. Already in 1914, Curtius and Franzen isolated *E*-2-hexenal from 600 kg of common hornbeam leaves and other tree species (Curtius and Franzen, 1914). However, it took another half of a century before researchers acknowledged the involvement of an ‘isomerization factor’ in the rearrangement from *Z*-3- to *E*-2-hexenal. In these early studies increased conversion rates to *E*-2-hexenal were reported in crude extract of cucumber fruits (Galliard et al., 1976) and soybean seeds (Takamura and Gardner, 1996), in macerated tea leaves (Hatanaka and Harada, 1973) and leaves of the leopard plant *Farfugium japonicum* (Hatanaka et al., 1976). HI activity has been partially purified from Alfalfa seedlings and cucumber fruits (Phillips et al., 1979; Noordermeer et al., 1999), but only very recently the gene responsible for the conversion from *Z*-3- to *E*-2-hexenal has been identified from red bell pepper fruits (Kunishima et al., 2016).

Here, we purified HI activity from cucumber fruits and identified the corresponding gene responsible for the rearrangement from *Z*-3- to *E*-2-hexenal. We identified in total four putative homologs in cucumber of which two possessed HI activity when expressed in *Escherichia coli* or transiently expressed in *Nicotiana benthamiana*. We furthermore identified tissue-specific expression patterns of the four putative homologs, tested wound-inducibility of the leaf-specific HI and determined kinetic parameters of the recombinant proteins of the two active cucumber HI homologs.

## Materials and Methods

### Plant Material and Growing Conditions

Cucumber seeds (*Cucumis sativus*, var. Kurios; E 31.2148) were kindly provided by Enza Zaden (Enkhuizen, NL^1^) and grown in the glasshouse with day/night temperatures of 23/18°C and a 16/8 h light/dark regime for 9–12 weeks. *N. benthamiana* plants were grown in the glasshouse for 3 weeks and then transferred to a climate room (25°C, 16/8 h light/dark, 70% humidity).

### Isomerase Activity Assays

Enzyme activity was determined by SPME-GC-ToF-MS or SPME-GC-QToF-MS. 200 μL of solution, either containing crude extract, purified fractions or purified recombinant proteins, were transferred to a 1.5 mL GC vial equipped with a 200 μL insert, and *Z*-3-hexenal in different concentrations was added to the solution. A volume of 200 μL was chosen to minimize the headspace and thus also the chance of *Z*-3-hexenal to volatilize. The GC vial was closed and gently shaken for 2 min (recombinant protein) or 5 min (collected fractions from partial purification). Subsequently, the liquid solution was transferred to a 20 mL SPME vial which was immediately closed with a Teflon lined crimp cap and incubated under moderate shaking for 1 or 5 min at 35°C prior to sampling.

### Analysis of Volatiles

Volatiles were initially analyzed by GC-ToF-MS (samples from the purification), until we obtained a new more sensitive GC-QTof-MS. In all cases volatiles were sampled with a Solid Phase Micro Extraction fiber (SPME; Carboxem/PDMS) for 10 min at 35°C.

### Analysis of Volatiles by GC-ToF-Ms

After sampling for 10 min the fiber was desorbed for 1 min in an Optic injector port (ATAS GL Int., Zoeterwoude, NL) which was constantly kept at 250°C. Compounds were separated on a DB-5 column (10 m × 180 μm, 0.18 μm film thickness; Hewlett Packard) in a 6890N gas chromatograph (Agilent, Amstelveen, NL) with a temperature program set to 40°C for 1.5 min, increasing to 250°C at 30°C per min and 250°C for an additional 2.5 min. Helium was used as carrier gas, with the transfer column flow set to 3 mL per minute for 2 min, and to 1.5 mL per minute thereafter. Mass spectra were generated by electron ionization with 70 eV electrons at 200°C and collected with a Time-of-Flight MS (Leco, Pegasus III, St. Joseph, MI, United States), with an acquisition rate of 20 scans per second.

### Analysis of Volatiles by GC-QToF-Ms

After sampling for 10 min the fiber was desorbed for 1 min in the injection port which was constantly kept at 250°C. Compounds were separated on HP-5 ms column (30 m × 250 μm, 0.25 μm film thickness; Agilent) in an Agilent 7890A gas chromatograph with a temperature program set to 40°C for 5 min, increasing to 140°C at a rate of 5°C per min, followed by increasing temperature to 250°C at a rate of 15°C per min and an additional 5 min at 250°C. Helium was used as the carrier gas with the transfer column flow set to 3 mL per minute and a flow rate of 1 mL per min thereafter. Mass spectra were generated by an Agilent 7200 accurate-mass quadrupole time-of-flight mass spectrometer, operating in electron ionization mode (70 eV) at 230°C and collected with an acquisition rate of 20 scans per second. volatiles were identified and quantified using standard solutions of *Z*-3-hexenal, *Z*-3-hexenol, *E*-2-hexenal, *E*-2-hexenol and (*E,Z*)-2,6-nonadienal (Sigma-Aldrich). (*Z,Z*)-3,6-nonadienal was synthesized from (*Z,Z*)-3,6-nonadienol (Ventos) as described below.

### Purification of (3*Z*):(2*E*)-Hexenal Isomerase Activity from Cucumber Fruits

All procedures (except for the FPLC runs at RT) were performed at 4°C.

*Step 1: Preparation of crude extract.* Two cucumber fruits were pealed and mixed in a blender. The crude extract was filtered through cheesecloth and Miracloth and subsequently diluted in ½ volume of buffer (20 mM MOPS pH 7.5, 0.2% Tween-20, 10 mM DTT). The solution was centrifuged at 10,000 *g* for 40 min at 4°C and the crude supernatant was pushed through a 0.45 μm filter, transferred to a new tube and stored at 4°C overnight.
*Step 2. Batch assay*, *cation exchange*. The crude supernatant (240 mL) was added to 1.5 g of carboxy methyl (CM) Sephadex C-25, which had been equilibrated in 50 mL MOPS (20 mM pH 7.5, 0.2% Tween-20) overnight. The suspension was gently shaken for 30 min at 4°C and centrifuged at 9,000 *g* for 25 min at 4°C. The supernatant was collected and tested for isomerase activity. The CM Sephadex pellet was resuspended in 200 mL of 200 mM NaCl and left on ice for 2 h. This solution was centrifuged at 9,000 *g* for 25 min at 4°C and the supernatant was collected and subsequently tested for isomerase activity. All isomerase activity was found in the (unbound) supernatant of the CM Sephadex.
*Step 3. Anion exchange chromatography*. To remove small particles we pushed the isomerase containing solution through a 0.22 μm filter and applied the filtered solution to a HiPrepQ XL 16/10 column (GE Healthcare) which had been equilibrated with starting buffer (50 mM MOPS pH 7.5). The adsorbed proteins were eluted with an increasing linear gradient of NaCl in the same buffer [0–1 M NaCl in 10 column volumes (CVs)] and a constant flow of 5 mL/min. Pools of 5 fractions (5 ml/fraction) were tested for isomerase activity. All active fractions were snap frozen and stored at -80°C.
*Step 4. Hydrophobic interaction chromatography*. The active fractions were pooled (6–20, approximately 67.5 mL; Supplementary Figure S1), diluted with 22.5 mL of 4 M (NH_4_)_2_SO_4_ and filtered (0.22 μm pore size). The filtered solution was applied to a HiPrep Phenyl HP 16/10 column which had been equilibrated with starting buffer [1 M (NH_4_)_2_SO_4_, 50 mM MOPS pH 7.5]. The adsorbed proteins were eluted with a linear gradient from 1 to 0 M (NH_4_)_2_SO_4_ in 50 mM MOPS pH 7.5 and a constant flow of 3 ml/min in 10 CVs. The active fractions (27–36, Supplementary Figure S1) were pooled and an equivalent of 50 μg of total protein was precipitated with 80% acetone. Two non-active fractions (13 and 14, Supplementary Figure S1) were taken along as negative controls. The precipitated pellets were re-suspended in 0.1 M Tris pH 7.6 in the same starting sample volume and used for SDS-PAGE and analysis with LC–MS [Supplementary Figure S1, *Step 5*; see below (Mass spectrometry analysis and identification) for details].
*Step 6. Gel filtration.* Since the above-mentioned purification steps did not suffice to identify a cucumber hexenal isomerase (Supplementary Table S1) we used 10 mL of the pooled active fractions (from step 4, Supplementary Figure S1), which had not been precipitated, for additional purification. The pooled active fractions were diluted in 0.5 mL 3 M NaCl and filter sterilized (0.22 μm pore size). The filtered solution was applied to a gel filtration column (Sephacryl S-300 High resolution, HiPrep 26/60; GE Healthcare) that had been equilibrated with buffer (50 mM MOPS, pH 7.5, 0.15 M NaCl). The column was run with a constant flow of 1.3 mL/min. Single fractions or a pool of fractions were tested for isomerase activity. All fractions were snap frozen and stored at -80°C.
*Step 7. Hydrophobic interaction chromatography*. The active fractions (10–12, Supplementary Figure S2) were pooled and the total of 15.75 mL was diluted with 5.25 mL 4 M (NH_4_)_2_SO_4_ and filtered (0.22 μm pore size). The filtered solution was applied to a HiTrap Phenyl HP (5 mL; GE Healthcare) column which had been equilibrated with starting buffer [1 M (NH_4_)_2_SO_4_, 50 mM MOPS pH 7.5]. The adsorbed proteins were eluted with a linear gradient from 1 to 0 M (NH_4_)_2_SO_4_ in 50 mM MOPS pH 7.5 and a constant flow of 3 ml/min in 10 CVs. Single fractions or a pool of fractions were tested for isomerase activity. Two mL of the active fractions (31–32, Supplementary Figure S2) and a pooled non-active fraction (28–30) were analyzed by LC–MS. All fractionations were performed with a FPLC (AKTA; GE healthcare) and protein elution was continuously monitored by UV absorption at 280 nm. Protein concentrations for step 1 till 4 were determined by Bradford assay using BSA for quantification. Because of the low protein concentrations after steps 6 and 7, we estimated the amount of total protein using 1 A_280_ = 1 mg/mL. Peak areas for this calculation were determined with ImageJ 1.50i using the original chromatograms of Supplementary Figure S2.

### Mass Spectrometric Analysis and Identification

#### Sample Handling Active versus Non-active Fractions

For the first comparative analysis (Purification part I, Supplementary Figure S1) about 3 μg of protein material from the pooled active and a non-active fraction were separated on 10% SDS-PAGE. No staining was used and the individual lanes were cut in 10 separate gel pieces from top to bottom. Each gel piece was digested in-gel with trypsin based on the method according to Shevchenko et al. (1996). The collected eluates after two separate 30 min extractions with 10 mM NH_4_HCO_3_ and one with 50% acetonitrile were freeze dried. The peptides were reconstituted in 20 μL 50% acetonitrile (ACN), 2% formic acid and stored at -20°C prior to analysis with LC–MS.

For the second comparison (Purification part II, Supplementary Figure S2) the liquid fractions were digested in solution according to an in-house trypsin based protocol. Alkylation before overnight digestion was done with iodoacetamide and a final concentration of 10% ACN was used to support the trypsin digestion. Digestion was stopped by adding trifluoric acid (TFA) with a final concentration of 1% and the sample was diluted to 3% acetonitrile. The peptides were collected by using a 2 μg capacity C18 ZipTips (Millipore) and the eluted peptides had a final volume of 10 μL 50% ACN, 0.1% TFA and were stored at -20°C before analysis with LC–MS.

#### Mass Spectrometric Analysis

An AmaZon Speed Iontrap MS/MS with a CaptiveSpray ion source (Bruker) coupled to an EASY-nLC II (Proxeon, Thermo Scientific) chromatographic system was used for the analysis. A fraction of the stored sample was dried in a speedvac and reconstituted in 6 μL 2% ACN, 0.1% TFA. Peptide samples were injected with a sample volume of 5 μL (Purification part I, Supplementary Table S1), 2 μL (Purification part II, Run 1, Supplementary Table S2) or 8 μL (Run 2, Supplementary Table S2) and separated with an eluent flow of 300 nL min^-1^ on an Acclaim PepMap100 (C18 75 μM 25 cm Dionex, Thermo Scientific) analytical column combined with an Acclaim PepMap100 pre-column (C18 100 μM 2 cm Dionex, Thermo Scientific) using a 30 min gradient of 0–50% ACN and 0.1% formic acid. Peptide precursor ions above a predefined threshold ion count were selected for low-energy collision-induced dissociation (CID) to obtain fragmentation spectra of the peptides. After processing the raw data with Data Analysis software (Bruker), the resulting mgf datafiles were used for database searching with Mascot software (Version 2.5.1) in an online available database of *C. sativus* (Csativus_122_protein.fa.gz; Phytozome v9.0^2^). Searches were simultaneously performed against a “common contaminants database”^3^ (compiled by Max Planck Institute of Biochemistry, Martinsried, Germany) to minimize false identifications. Mascot search parameters were a fixed modification of carbamidomethyl for cysteine, variable modification of oxidized methionine and trypsin with the allowance of one missed cleavage and peptide charge state +2, +3, and +4. Peptide and MS/MS mass error tolerances were 0.3 Da for ESI-Trap.

### Cloning and Construct Design

The cDNA sequences of Cucumber Cs033080, Cs033090, Cs078390, Cs240840, and Cs387820 were obtained from Phytozome^2^ (accession numbers: Cucsa.033080.1, Cucsa.033090.1, Cucsa.078390.1, Cucsa.240840.1 and Cucsa.387820.1, Supplementary Table S3). The ORFs of four candidates were cloned from a mix of cucumber leaf, tendril, and petiole cDNA in the pJET1.2/blunt vector (Thermo Fisher) and verified by sequencing. Unfortunately we were unable to clone candidate Cs387820. Primers containing the attB1 and attB2 Gateway recombination sites were used to amplify all ORF sequences. The resulting PCR products were recombined with the Gateway vector pDONR207 (Clontech) using BP Clonase II (Thermo Fisher). All constructs were verified by PCR and sequencing prior to LR-reaction with the destination vector. For the transient assay in *N. benthamiana* leaves the cDNA clones were introduced into the destination vector pK2GW7 under control of the 35S CaMV promoter^4^. For expression of recombinant protein in *E coli* the cDNA clones were introduced into the destination vector pGEX-KG-GW (Dhonukshe et al., 2010). LR reactions were performed with LR Clonase II (Thermo Fisher) and the resulting clones were confirmed by PCR and sequencing.

### Transient Expression of Protein in *Nicotiana benthamiana* Leaves

Agro-infiltrations were performed with 4 week-old *N. benthamiana* plants. *Agrobacterium tumefaciens* GV3101 (pMP90) cultures carrying the HI-candidate constructs (pK2GW7: 35S-Cs033090, 35S-Cs033080, 35S-Cs240840, 35S-Cs078390) or a construct carrying only GFP (pK7WGF2.0) (Karimi et al., 2005) were grown overnight from a single colony and diluted in infiltration buffer [¼ (v/v) LB medium, ¼ (v/v) sterile H_2_O, ½ (v/v) 2x MS medium in 10 mM MES pH 5.6, 20 mM glucose, 10% (w/v) sucrose, and 200 μM acetosyringone] to an OD_600_ of 0.6. To suppress gene silencing each construct was co-infiltrated in a 1:1 ratio with an *A. tumefaciens* GV3101 (pMP90) strain that carried the pBIN61 vector to express the P19 suppressor (Voinnet et al., 2003). Six leaves of three plants were infiltrated for each HI-construct. Different areas of each leaf were infiltrated with two control samples [infiltration buffer and 35S-GFP (pK7WGF2.0)] and one of the HI-constructs. Five, six and seven days after infiltration leaf disks with a diameter of 2 cm were sequentially collected per plant for each infiltration area, and leaf material per plant and construct was pooled prior to analysis. The collected leaf disks were immediately wounded five times with a riffled forceps and placed in a 20 mL SPME vial which was immediately closed with a Teflon lined crimp cap and measured with GC-QToF-MS. Volatiles were sampled with a Solid Phase Micro Extraction fiber (SPME; Carboxem/PDMS) for 10 min at 35°C and volatiles were analyzed as described above. Since it was impossible to measure all samples on 1 day we measured biological replicates on three consecutive days.

### Production of Recombinant Protein

For the production of recombinant protein the constructs were transformed into *E. coli* C41 (DE3) cells. A single colony was inoculated in 2-YT medium (Tryptone 16 g/l, Yeast extract 10 g/l, NaCl 5 g/l). The next day, the culture was diluted 1:30 in 150 mL of 2-YT medium and grown at 37°C until an OD_600_ of approximately 0.9. Protein expression was induced with 1 mM Isopropyl-β-D-thiogalactoside and the cultures were incubated at 20°C for 22 h with 200 rpm agitation. Cells were harvested by centrifugation at 5,000 *g* for 15 min and pellets were snap frozen in liquid nitrogen, and stored at -80°C. The harvested cells were re-suspended in extraction buffer [20 mM Tris buffer (pH 7.5), 100 mM NaCl, 5 mM EDTA, 1 mM EGTA, 5 mM DTT] containing lysozyme (1 mg/mL) and 10 mM Complete Protease Inhibitor Cocktail (Roche Amersham). Cells were incubated on ice for 30 min and subsequently lysed by sonicating six times for each 20 s, and crude protein extracts were centrifuged at 10,000 *g* for 15 min. Five mL of the supernatant was pushed through a 0.22 μm filter and the filtered solution was applied to a 1 mL GSTrap FF column (GE Healthcare) which had been equilibrated with starting buffer (1x PBS). The flow rate was kept at 0.2 mL/min during sample loading and was increased to 1 mL/min afterward. Proteins were eluted stepwise with elution buffer (50 mM Tris-HCl pH 8, 10 mM reduced glutathione, 10 mM Complete Protease Inhibitor Cocktail), which was continuously monitored by UV absorption at 280 nm. Purified recombinant proteins (Supplementary Figure S5) were tested for isomerase activity, 10% glycerol was added to the protein and aliquots were snap-frozen at -80°C.

### Kinetics

In order to determine the kinetic parameters of the two active HIs, Cs033090 and Cs078390, purified GST-tagged proteins were used (see above for details of the assay). Purified GST-tagged protein (∼63 kDa; 6 ng for 033090 and 420 ng for 078390) was diluted in reaction buffer [10 mM MOPS, pH 7.5, 2 μL BSA (10 mg/mL)] to a final volume of 200 μL. The substrate *Z*-3-hexenal was added to the reaction mixture in a concentration range of 100–2500 μM and the mixture was incubated for 2 min at RT while gently shaking. To stay within the linear range of the instrument (GC-QToF-MS) an aliquot (4–100 μL) of the reaction mixture containing an equivalent of 50 μM *Z*-3-hexenal was transferred to a 20 mL SPME vial, immediately closed and volatiles were absorbed onto the fiber for 10 min. The fiber was subsequently injected into the GC and volatiles were analyzed as described above. To determine the conversion from *Z*-3-hexenal to *E*-2-hexenal we first calculated the sum of aldehydes (*Z*-3-hexenal + *E*-2-hexenal) measured by SPME, taking the response factors of each compound into account. We subsequently calculated the percentage of *E*-2-hexenal and subtracted the non-enzymatic conversion from this value. Final concentrations of *E*-2-hexenal were then re-calculated by taking the initial concentration of Z-3-hexenal used for the assay into account. K_m_ and K_cat_ values were determined from Lineweaver–Burk Plots (*n* = 3 for HI-1, *n* = 4 for HI-2).

### (*Z,Z*)-3,6-Nonadienal Synthesis and Substrate Testing

The aldehyde (*Z,Z*)-3,6-nonadienal was synthesized from (*Z,Z*)-3,6-nonadienol (Ventos, ^5^Barcelona, Spain) by oxidation with *o*-iodoxybenzoic acid as described in Sasaki et al. (2011). To test whether the two active HIs, Cs033090 and Cs078390, are able to use also (*Z,Z*)-3,6-nonadienal as substrate we measured the conversion of (*Z,Z*)-3,6-nonadienal to (*E,Z*)-2,6-nonadienal by SPME-GC-QToF-MS as described above using 2 μg of purified GST-tagged protein.

### Quantitative Real-Time PCR

For tissue-specific expression analysis we harvested material from three 9 week-old cucumber plants. We used the third youngest leaf [leaf; **Figure 5** (c)] and its petiole [petiole (d)], the tendrils of the three youngest leaves [tendril (e)] and two fruits per plant, which were pealed to separately test skin and pealed fruit [fruit (a) and skin (b)]. Plant material was flash frozen in liquid nitrogen and stored at -80°C until use.

For the time-course experiment we used four plants per time point. The first fully expanded true leaf of 2 week-old cucumber plants was wounded with a pattern wheel to punch five rows of holes on each side of the midrib. After the indicated time (between 30 min and 6 h) the treated leaves were harvested, flash frozen in liquid nitrogen and stored at -80°C until use. Samples from untreated plants were used as controls.

Total RNA was extracted from ground plant material using TRIzol Reagent (Thermo Fisher) according to the manufacturer’s protocol, and DNase treated (TURBO DNase kit, Ambion). cDNA was synthesized from 1 μg of total RNA using the RevertAid kit (Fermentas). Quantitative real-time PCR (ABI 7500 Real-Time PCR System; Applied Biosystems) was done with cDNA equivalents of 10 ng total RNA using the EVAGreen Real-Time PCR Mastermix (Biotium) and gene-specific primers (Supplementary Table S4). Specificity was verified by dissociation analysis and expression of cucumber *Actin-like7* gene was used for normalization (Shi et al., 2015). Primer efficiencies were calculated by analysis of amplification curves of a cDNA dilution range. Three to four biological replicates were analyzed individually.

### Statistical Analysis

Statistics were done with IBM SPSS Statistics 22. Data were compared using analysis of variance (ANOVA) followed by a Scheffé *post hoc* test.

## Results

### Purification of a (3*Z*):(2*E*)-Hexenal Isomerase from Cucumber Fruits

(3*Z*):(2*E*)-hexenal isomerase activity has been described for several plant species (Phillips et al., 1979; Takamura and Gardner, 1996; Noordermeer et al., 1999), but the underlying gene responsible for the conversion from *Z*-3-hexenal to *E*-2-hexenal had, until recently (Kunishima et al., 2016), not been identified. In order to identify such hexenal isomerase (HI) we purified proteins from pealed cucumber fruits and used a GC–MS based assay to drive the fractionation of enzymatic activity. Crude cucumber extract was sequentially fractionated by cation exchange, anion exchange, and hydrophobic interaction chromatography (HIC; Supplementary Figure S1). However, the purification steps only led to a moderate increase in specific activity (**Table 1**). This was reflected by the high number of possible candidates identified by LC–MS/MS from in-gel trypsin-digested slices of pooled active and non-active fractions that had been separated on SDS-PAGE (Supplementary Figure S3). Since our purification strategy did not suffice to identify a cucumber HI (Supplementary Table S1) we proceeded with the purification.

**Table 1:** Summary of (3*Z*):(2*E*)-hexenal isomerase purification from cucumber fruits^a^.

Step	Description	Total protein (mg)	Total activity (units)^c^	Specific activity (units/mg)	Purification (fold)	Yield (%)
1	Crude extract	56.2^b^	2103.9	37.4	1.0	100
2	CM-Sephadex	45.9^b^	992.5	21.6	0.6	47.2
3	Sepharose-Q column	7.7^b^	657.6	85.2	2.3	31.3
4	Phenyl Sepharose column	4.9^b^	458.5	93.8	2.5	21.8
6	Sephacryl S-300 column	0.04^d^	86.9	2421	64.7	4.1
7	Phenyl-Sepharose column	0.0006^d^	24.2	43472	1161	1.2

^a^*Detailed information about the purification steps can be found in Supplementary Figures S1, S2.*

^b^*Protein concentration determined by Bradford assay using BSA as standard.*

^c^*1 unit causes 50% conversion to *E*-2-hexenal in 5 min.*

^d^*Protein concentration was estimated using 1 A_280_ = 1 mg/mL*.

Pooled active fractions were sequentially fractionated on gel filtration and HIC columns (Supplementary Figure S2). This procedure overall led to a 1161-fold purification with a 1.2% recovery (yield; **Table 1**). The pooled final active (31 and 32) and non-active (27–29) fractions were digested with trypsin and analyzed by LC–MS/MS, which resulted in a list of candidate proteins that were either enriched or solely present in the active fraction (Supplementary Table S2). While the two most abundant proteins identified from the active fractions, arginase (Cs362680) and protein-disulfide isomerase (Cs188190), are well-described enzymes involved in the release of nitrogen from arginine (Chen et al., 2004) and the correct folding of proteins (Cho et al., 2011), respectively, the third most abundant candidate, Cs033090, was assigned as a cupin-like protein. This candidate gave a very high protein coverage score (Supplementary Table S2 and Figure S4; 59% AA coverage) and was clearly enriched in the active fractions of both runs compared to the non-active fractions (Run II: protein score of 1302 versus 35).

### The (3*Z*):(2*E*)-Hexenal Isomerase Is a Cupin-Like Protein

The superfamily of cupins is a functionally very diverse group of proteins with a putative β-barrel shape. The family consists of enzymatic and non-enzymatic members with either one, two, or even more cupin domains which have been described for prokaryotes as well as eukaryotes (Dunwell et al., 2004). The main characteristic of the cupin domain is a two-motif structure consisting of conserved motif 1 [G(X)_5_HXH(X)_3_,_4_E(X)_6_G] and conserved motif 2 [G(X)_5_PXG(X)_2_H(X)_3_N] with a variable intermotif spacing of 15 to approximately 50 amino acids (Dunwell et al., 2001).

**FIGURE 1:**
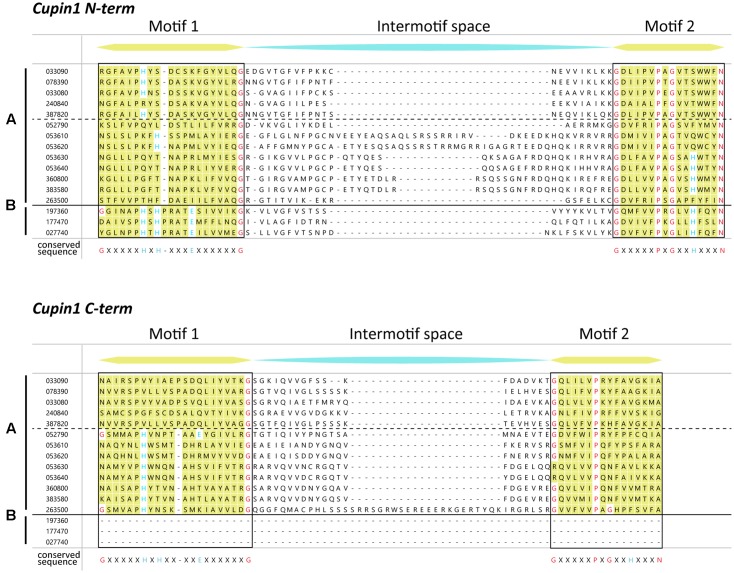
Multiple sequence alignment of the two conserved motifs of putative cupin proteins from cucumber containing a Pfam00190 (cupin1) family. The two conserved motifs of cupins are denoted in yellow, with highly conserved residues in red (conserved structurally important residues) and blue (conserved active-site residues). Sequences above the dotted line are proposed to be homologs of Cs033090, the putative hexenal isomerase. The sequences are divided according to the number of cupin domains [one cupin domain (B); two cupin domains (A)]. The conserved sequence for each motif is given below the alignment [motif 1: G(x)_5_HxH(x)_3,__4_E(x)_6_G; motif 2: G(x)_5_PxG(x)_2_H(x)_3_N]. Cucumber sequences with a Pfam00190 were retrieved from Phytozome and incomplete sequences (two sequences) were removed prior to analysis. Alignment was done with MegAlign Pro using the clustal omega algorithm with default settings. N-term, N-terminal; C-term, C-terminal.

**FIGURE 2:**
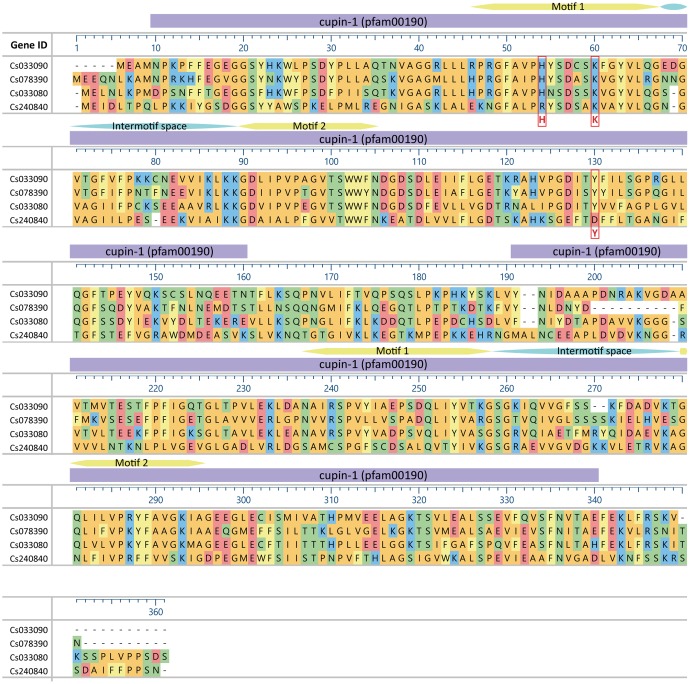
Alignment of hexenal isomerase (HI) proteins from cucumber. The position of amino acids HKY of the catalytic site according to Kunishima et al. (2016) are marked with a red box. Conserved domains are indicated on top as purple bars, conserved motifs and interspace motifs in yellow and blue, respectively. Amino acids are colored according to their side chain chemistry. Alignment was done with MegAlign Pro using the clustal omega algorithm with default settings.

**FIGURE 3:**
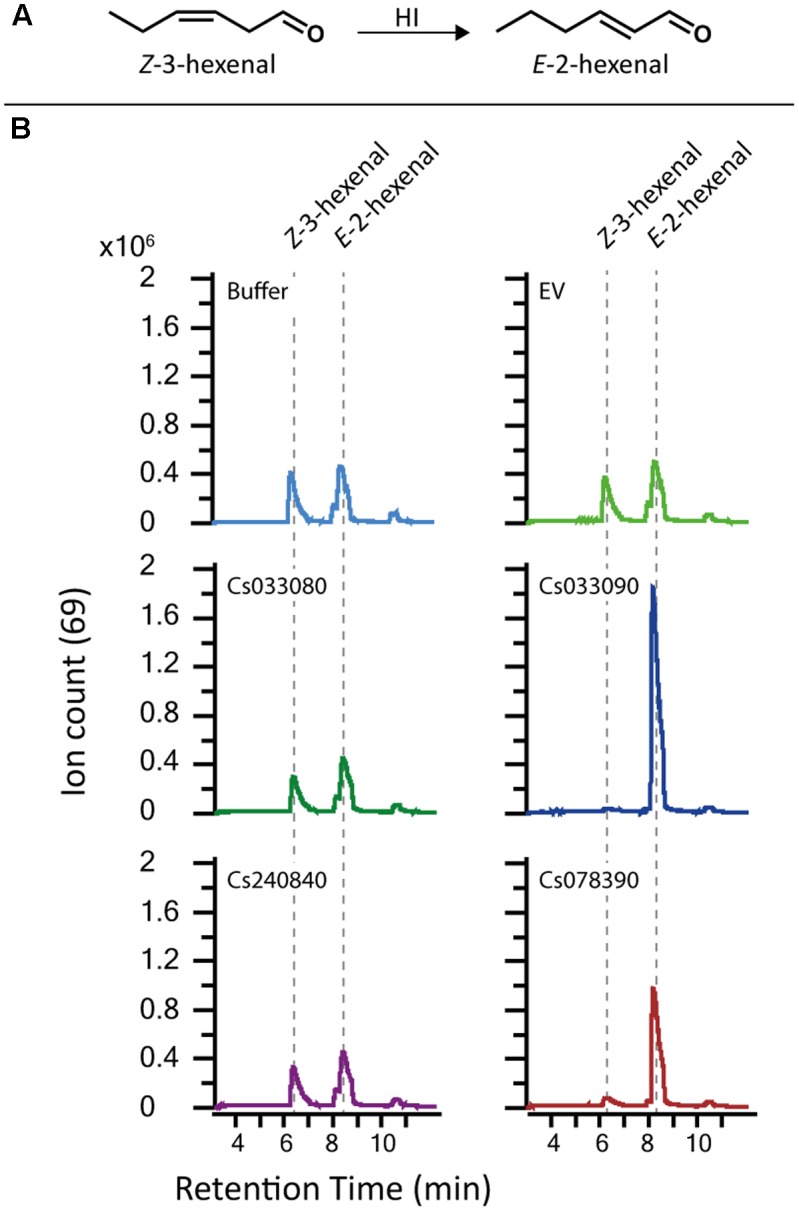
Recombinant proteins of two HI cucumber homologs have (3*Z*):(2*E*)-hexenal isomerase activity. **(A)** Structures of the GLVs *Z*-3-hexenal and *E*-2-hexenal. **(B)** GLV-related GC-QTOF-MS extracted ion chromatogram (Ion 69) as output of the SPME-guided assay for measuring (3*Z*):(2*E*)-hexenal isomerase activity. Equal amounts of purified recombinant protein (2 μg each) were tested for isomerase activity. Buffer and recombinant protein of GST (EV) were used as control. Only Cs03390 and Cs078390 but not Cs033080 and Cs240840 showed activity. *Z*-3-hexenal was used as substrate. HI; hexenal isomerase.

To find homologs of the cupin-like protein Cs033090 from *C. sativus* we collected AA sequences possessing the same conserved domain (Pfam 00190) as the candidate HI and aligned the two-motif sequences of all *in silico* putative homologs. We identified three monocupins and 13 bicupins with the same conserved domain. Four of the bicupins showed a high sequence identity to the candidate HI (Cs033090) within (**Figure 1**) and outside of (**Figure 2**) the conserved motifs. The highest similarity was detected between Cs033090 and Cs078390, these proteins were 63% identical. Sequences of Cs033080 and Cs240840 shared only 60 and 38% identity with the candidate HI (Cs033090). To determine whether the candidate HI and its putative homologs possess (3*Z*):(2*E*)-hexenal isomerase activity we cloned the corresponding cDNAs of four (unfortunately we were unable to clone the putative HI Cs387820) of the five candidates and expressed their recombinant protein in *E. coli*. The purified recombinant protein of the initial candidate Cs033090 indeed possessed isomerase activity (from hereon called CsHI-1), as did the recombinant protein of the nearest homolog (Cs078390; now called CsHI-2) (**Figure 3**). Activity assays with both other homologs (Cs033080 and Cs240840; from hereon called CsHI-like 1 and CsHI-like 2, respectively) showed conversion rates from *Z*-3-hexenal to *E*-2-hexenal that did not differ from controls (**Figure 3**).

### Determination of Kinetic Parameters of the Active HIs

The determination of kinetic parameters of the recombinant proteins rCsHI-1 and rCsHI-2 for *Z*-3-hexenal revealed that K_m_ values of both recombinant enzymes were comparable (rCsHI-1, 0.6 ± 1.3 mM^-1^; rCsHI-2, 0.2 ± 0.05 mM^-1^). However, the turnover rate (K_cat_) of rCsHI-1 was 90-times higher than that of rCsHI-2 (638 ± 134 s^-1^ and 7.6 ± 1.6 s^-1^ mM^-1^) leading to a 30-fold higher K_cat_/K_m_ ratio.

### Transient Expression of CsHIs in *N. benthaminana* Changes the GLV Profile of Plants

**FIGURE 4:**
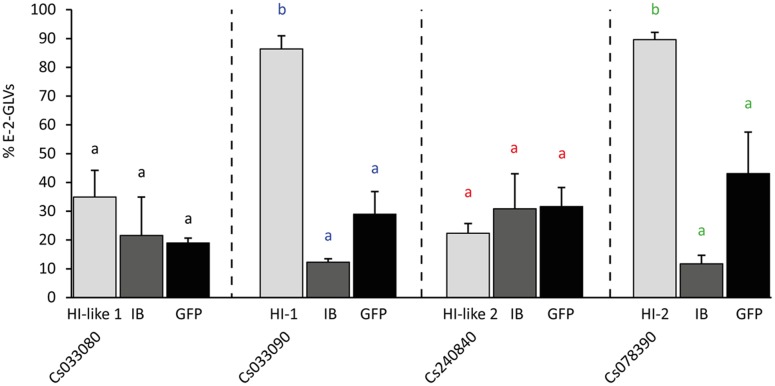
Relative emission of *E*-2-GLVs in *Nicotiana benthamiana* leaves transiently expressing HI cucumber homologs. Two leaves per plant were infiltrated with *Agrobacterium tumefaciens* harboring one of the 35S-HI (hexenal isomerase) constructs or a 35S-GFP (GFP) construct or only the infiltration buffer (IB). From the equally treated areas of the two leaves per plant, leaf disks were punched out and artificially wounded. GLV emission (*Z*-3-hexenal, *Z*-3-hexenol, *E*-2-hexenal, and *E*-2-hexenol) of the two leaf disks was measured immediately after wounding and the percentage of *E*-2-GLVs was calculated. Statistics were done for each construct separately compared to infiltration buffer and GFP (letters with same color): different letters indicate significant differences between the treatments (univariate ANOVA, Cs033080: *F*_2,6_ = 0.82, *p* = 0.48; Cs033090: *F*_2,6_ = 54.07, *p* < 0.001; Cs240840: *F*_2,6_ = 0.39, *p* = 0.69; Cs078390: *F*_2,6_ = 20.74, *p* < 0.001, followed by a Scheffé *post hoc* test).

Plants produce considerable amounts of GLVs as a response to biotic and abiotic stress (Scala et al., 2013a). In order to test how expression of a cucumber-derived HI might change the GLV profile of plants that possess no substantial HI-activity by themselves we transiently expressed the four HI homologs in *N. benthamiana* and determined the GLV profile of infiltrated leaves after mechanical damage. Headspace of treated leaf disks was collected immediately after wounding for a period of 10 min. Within this timeframe plants release mainly aldehydes and alcohols (Fall et al., 1999). While wounded leaf disks of plants expressing CsHI-1 and CsHI-2 emitted almost exclusively *E*-2-GLVs (86 and 90%, respectively) expression of CsHI-like 1 and 2 did not cause a significant increase in *E*-2-GLV levels compared to control treatments (**Figure 4**).

### HI Homologs Have Different Patterns of Expression

**FIGURE 5:**
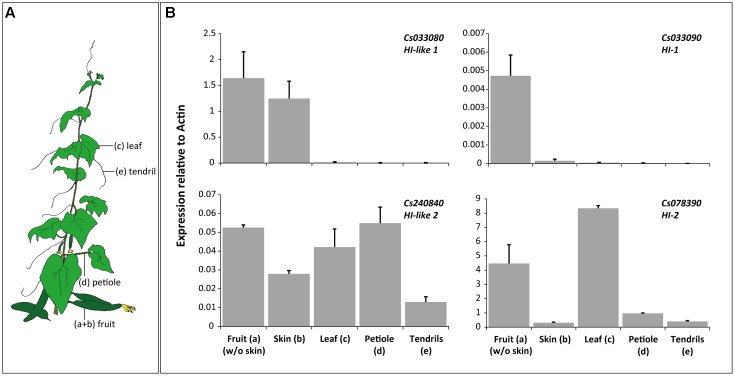
Transcript abundance of HIs in different tissues of 9 week old cucumber plants. **(A)** Drawing of a cucumber plant illustrating which plant tissues were used for qRT-PCR. **(B)** Mean (+SEM) relative transcript abundance of the four HIs in peeled fruits (a), their skin (b), leaves (c), petioles of the same leaves (d) and tendrils (e).

To determine whether the four HI homologs exhibit tissue specific transcript levels we collected plant tissue from leaves, petioles, tendrils, and fruits (**Figure 5A**). While CsHI-1 (Cs033090) was specifically expressed in cucumber fruits, HI-2 (Cs078390) was expressed in both, reproductive (fruits) and vegetative (leaves) tissue (**Figure 5B**). Interestingly, expression of both HIs was stronger in the flesh than in the skin of the fruit. This clear distinction between flesh and skin in HI transcript levels was not detectable for CsHI-like 1 (Cs033080) which was only expressed in cucumber fruits. We did not observe a clear tissue-specific expression pattern for CsHI-like 2 (Cs240840; **Figure 5B**).

**FIGURE 6:**
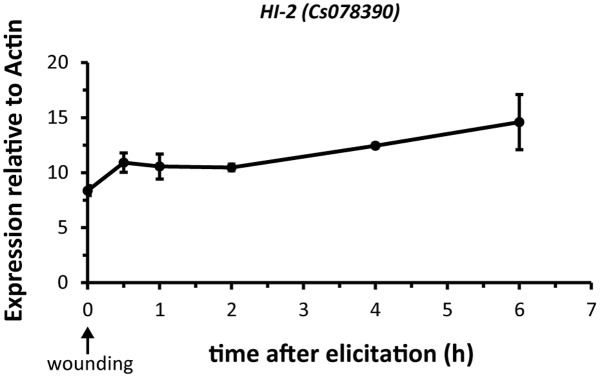
Wound-inducibility of *HI-2* in cucumber leaves. Mean (± SEM) expression of *HI-2* relative to actin. The first fully expanded leaf was harvested from un-wounded (0 h) and mechanically wounded 2-weeks old cucumber plants. Individual leaves from four independent plants per time point (*n* = 4) were harvested at each indicated time after elicitation. The abundance of *HI-2* transcript was analyzed by qRT-PCR and normalized to a reference gene (actin).

Plants release high amounts of GLVs from their vegetative tissue upon wounding (Scala et al., 2013a). To test whether an increase in GLVs coincides with an increase in transcript levels of *HI-2*, which is expressed in leaves (**Figure 5B**), we harvested leaves from non-wounded and mechanically wounded cucumber plants. *CsHI-2* transcript levels slightly increased 30 min after the treatment and stayed on this slightly elevated level the following 5 h (**Figure 6**).

### (*Z,Z*)-3,6-Nonadienal Can Be Used As Substrate by Both Hexenal Isomerases

**FIGURE 7:**
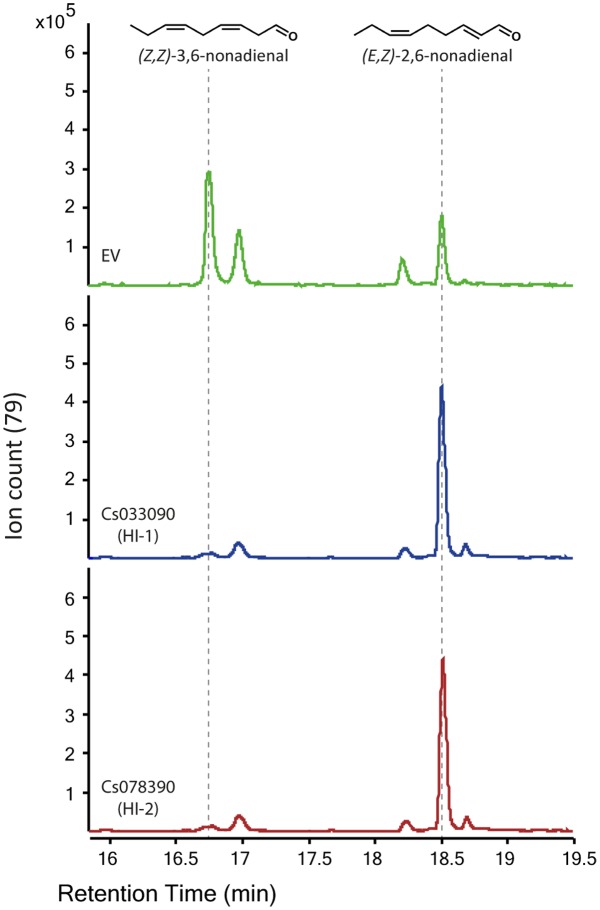
Recombinant proteins of the two active HIs from cucumber can also use (*Z*,*Z*)-3,6-nonadienal as substrate. GLV-related GC-QTOF-MS extracted ion chromatogram (Ion 79) as output of the SPME-guided assay for measuring (3*Z*):(2*E*)-enal isomerase activity. Equal amounts of purified recombinant protein (2 μg each) of the two active HIs (Cs033090 and Cs078390) were tested. Recombinant protein of GST (EV) was used as control. (*Z,Z*)-3,6-nonadienal was used as substrate.

HI-1 has initially been purified from cucumber fruits. Also, transcript analysis revealed that this HI is solely expressed in the flesh of cucumbers. However, fruits contain only small amounts of *E*-2-hexenal while other *E*-2-alkenals, including *E*-2-nonenal and (*E,Z*)-2,6-nonadienal have been detected in much higher quantities in this tissue (Wei et al., 2016). Since (*Z,Z*)-3,6-nonadienal has been shown to serve as substrate for a red bell pepper HI (Kunishima et al., 2016) we tested whether this was also the case for the two active cucumber HIs. Indeed, recombinant proteins of both HI-1 and HI-2 were able to convert (*Z,Z*)-3,6-nonadienal to (*E,Z*)-2,6-nonadienal (**Figure 7**). These results indicate that *Z*-3-alkenals with different chain length and quantities of double bonds can be used as substrate by CsHI-1 and CsHI-2.

## Discussion

*E*-2-hexenal is a commonly occurring compound in the volatile bouquet of stressed plants and plays an important role in transferring information to plants and insects either as a single molecule (Farag and Paré, 2002; James, 2005; Kessler et al., 2006) or within a complex volatile mixture (Allmann and Baldwin, 2010; Allmann et al., 2013). While most biosynthetic enzymes upstream of *E*-2-hexenal have been studied extensively (Scala et al., 2013a; Mwenda and Matsui, 2014; ul Hassan et al., 2015), detailed information about the enzyme responsible for the conversion from *Z*-3- to *E*-2-hexenal had, until recently (Kunishima et al., 2016), been largely missing. In this study we have successfully identified two (3*Z*):(2*E*)-hexenal isomerases (HIs) from cucumber fruits with different catalytic efficiencies for *Z*-3-hexenal. These hexenal isomerases were also able to convert (*Z,Z*)-3,6-nonadienal to (*E,Z*)-2,6-nonadienal, the latter representing the main flavor volatile of cucumber fruits (Huang et al., 2009). Overexpression of those HIs drastically changed the GLV profile of *N. benthamiana* plants.

Recently an HI has also been identified from bell pepper fruits and *Z*-3- to *E*-2-hexenal converting activity was confirmed by heterologous expression of a bell pepper HI and orthologous HIs of other plant species. Through point mutations of single amino acids H54, K60 and Y128, which led to a loss-of-function of recombinant protein of the bell pepper HI, Kunishima et al. (2016) identified a putative catalytic site (catalytic HKY) which was considered as an important prerequisite for the enzymatic function of HIs. The presence or absence of this catalytic HKY was subsequently used to identify putative plant HIs with or without *Z*-3- to *E*-2-hexenal converting activity and they were referred to as HIs and HI-like proteins, respectively. In this way three putative HIs and one HI-like protein from cucumber were identified by Kunishima et al. (2016). However, while we identified the same 4 sequences in cucumber (**Figures 1**, **2**), heterologous expression of these proteins revealed that only two of the three putative HIs with a catalytic HKY possessed HI-activity (**Figures 3**, **4**). The third putative HI (Cs033080) was unable to convert *Z*-3- to *E*-2-hexenal, also when transiently overexpressed in *N. benthamiana* (**Figure 4**) and we thus referred to it as CsHI-like 1. The fact that this protein is unable to convert *Z*-3-hexenal to its *E*-2-isomer is rather surprising, since Kunishima et al. (2016) reported that recombinant protein of this putative HI possessed (3*Z*):(2*E*)-hexenal isomerase activity. The RefseqID that was given for the cucumber HI tested for isomerase activity (XP_011651276.1) matches with the sequence of our CsHI-like 1 (Cs033080). However, the primers they used to clone the putative HI do not align to CsHI-like 1, but to CsHI-1 (Cs033090) which, also in our case, exhibits (3*Z*):(2*E*)-hexenal isomerase activity. We thus believe that CsHI-1 (Cs033090) instead of CsHI-like 1 (Cs033080) was cloned and heterologously expressed by the Japanese group. However, the question remains, why does CsHI-like 1 (Cs033080) show no activity although it possesses the catalytic HKY? Seed storage globulins are bicupins which are very closely related to the HIs. These proteins have lost their enzymatic activity most likely by losing three of the four metal-binding H and E residues in both of the two domains (Dunwell et al., 2004; **Figure 1**). Interestingly this loss of metal-binding residues did not lead to a loss of enzymatic activity for the two active HIs (Cs033090 and Cs078390; **Figures 1**, **3**, **4**). Further research will reveal whether other parts within or outside of the two motif sequence are equally important for maintaining enzymatic activity, whether CsHI-like 1 (Cs033080) might be active toward other, structurally related compounds or whether other changes in the sequence, e.g., the C-terminal extension (**Figure 2**), might have led to an inactivation of this protein.

Affinity tags are helpful tools to purify recombinant protein. However, they can also affect the biochemical properties of the target protein leading to, amongst others, inhibition or alteration of enzyme activity (Arnau et al., 2006; Costa et al., 2014). For our activity assay we used recombinant protein of which the GST-tag had not been removed prior to analysis. This might have altered the interaction between enzyme and substrate and thus might have influenced the outcome of the kinetic parameters. Although we cannot fully exclude this possibility, it is rather unlikely that the GST-fusion had a considerable effect on enzyme activity; recombinant protein of HIs from several plant species of which the His-tag had been removed prior to analysis resulted in very similar kinetic parameters (**Table 2**) ranging, e.g., from 0.2 to 1.78 mM for K_m_ values and from 27.7 to 521 s^-1^ for K_cat_ values (Kunishima et al., 2016).

**Table 2:** Kinetic parameters of recombinant cucumber (3*Z*):(2*E*)-hexenal isomerases^a^.

	K_cat_ (s^-1^)	K_m_ (mM)	K_cat_/K_m_ (s^-1^ mM^-1^)
HI-1	683 ± 134	0.6 ± 0.13	1129 ± 223
HI-2	7.6 ± 1.6	0.2 ± 0.05	37.7 ± 2.1

^a^*Z-3-hexenal has been used as substrate*.

Cucumber possesses 23 different *LOX* genes (Huang et al., 2009) which are divided into type 1 (9-LOX) or type 2 (13-LOX) LOXs (Yang et al., 2012). This notable expansion of LOXs in the cucumber genome and the fact that cucumber has two tandem *HPL* genes (Huang et al., 2009) that can cleave both, 9- and 13-HPOs (Matsui et al., 2000; Wan et al., 2013), suggests that 9- and 13-HPL cleavage products play an important role in physiological and/or environmentally regulated processes. The main HPL-cleavage products in cucumber plants are the C9-aldehydes, *Z*-3-nonenal and (*Z*,*Z*)-3,6-nonadienal, which are formed from linoleic acid and α-linolenic acid, respectively, and the C6-aldehyde, *Z*-3-hexenal (Matsui et al., 2000). However, due to the high HI-activity in cucumber these *Z*-3-aldehydes can be quickly converted to its *E*-2-isomers (Matsui et al., 2006). By testing the substrate specificity of partially purified HI from cucumber Phillips et al. (1979) revealed that the enzyme was able to convert *Z*-3-aldehydes with different chain lengths but showed highest efficiencies with *Z*-3-hexenal and *Z*-3-nonenal. Here we have shown that also (*Z*,*Z*)-3,6-nonadienal can be used as substrate by both HIs to form (*E*,*Z*)-2,6-nonadienal (**Figure 7**). Interestingly, (*E*,*Z*)-2,6-nonadienal was detected in high levels in the reproductive tissues of cucumber, while *E*-2-hexenal, although ubiquitously present in all tested tissues, was mainly produced in the vegetative tissues, including leaves and roots (Wei et al., 2016). It is thus likely that CsHI-1 (Cs033090), which is specifically expressed in the flesh of cucumber fruits, is mainly responsible for the rearrangement of the 9-HPL cleavage product from α-linolenic acid, (*Z*,*Z*)-3,6-nonadienal, whereas CsHI-2 (Cs078390), which is expressed in fruits and leaves, represents the main HI responsible for the conversion of *Z*-3-hexenal to *E*-2-hexenal.

While we have identified the enzyme responsible for the conversion from *Z*-3- to *E*-2-aldehydes the question remains what the main function of this increased conversion is for plants. Recent research revealed that an increased conversion from *Z*-3- to *E*-2-GLVs can be beneficial for herbivore attacked plants by attracting natural enemies of the herbivores (Allmann and Baldwin, 2010) and by deterring gravid female moths (Allmann et al., 2013). Our results show, in contrast to previous results from Kunishima et al. (2016), that wounding only slightly increased the transcript levels of the leaf-specific *HI-2* (**Figure 6**). Future research will reveal whether there is a tight correlation between the amount of damage applied to the plant and HI expression, whether herbivore-specific factors are needed to amplify the wound-induced increase in transcript levels, whether HIs are post-transcriptionally regulated or whether they are constitutively expressed in leaves. In any case, there are also other, not mutually contradictory, explanations why plants possess (3*Z*):(2*E*)-hexenal isomerases: one possibility is that plants produce *E*-2-aldehydes in their fruits and leaves as a protection against microbial invaders; C6- as well as C9-aldehydes have bactericidal (Nakamura and Hatanaka, 2002; Cho et al., 2004) and antifungal (Hamiltonkemp et al., 1992; Matsui et al., 2006) properties and although the current literature is not conclusive on whether *E*-2-aldehydes are more effective than *Z*-3-aldehydes (Nakamura and Hatanaka, 2002; Kishimoto et al., 2005; Prost et al., 2005; Tajul et al., 2012) the presence of an α,β-unsaturated carbonyl moiety in *E*-2-aldehydes and the resulting high reactivity with nucleophilic atoms (Alméras et al., 2003) might enhance the antimicrobial properties of these molecules. Interestingly, the presence of an α,β-unsaturated carbonyl group was not essential for several C6- and C9-compounds to serve as fungicidal agent against *Botrytis cinerea* and *Fusarium oxysporum* (Matsui et al., 2006). Clearly, much more work is needed to understand the physiological function of HIs for plants and the role they might play in plant–insect and plant–pathogen interactions. The engineering and characterization of plants with either reduced or no HI-activity will help to unravel the main function of this enzyme in plants.

Hexenal isomerase is by far one of the less investigated enzymes of the GLV biosynthesis pathway. Our results will not only impact future research on plant-biotic interaction, but might lead to advances in applications as GLVs and their derivatives play pivotal roles in the food and aroma industry.

## Author Contributions

SA, ES, and RS conceived the study, SA, ES, HD, and LS performed experiments, SA, ES, and HD analyzed the data, SA wrote the manuscript, ES, RS, HD, MH, CdK, and JvM read and commented on the manuscript.

## Conflict of Interest Statement

The authors declare that the research was conducted in the absence of any commercial or financial relationships that could be construed as a potential conflict of interest.

## Footnotes

1 http://www.enzazaden.nl/

2 https://phytozome.jgi.doe.gov/pz/portal.html

3 http://www.matrixscience.com/help/seq_db_setup_contaminants.html

4 http://www.vib.be

5 https://www.ventos.com

## Abbreviations

GLVs: green leaf volatiles
HI: hexenal isomerase
HPL: hydroperoxide lyase
13-HPOs: 13-hydroperoxides
LOX: lipoxygenase.
