# Supplementary material for: Identification and Characterization of (3Z):(2E)-Hexenal Isomerases from Cucumber
Source: Front Plant Sci. 2017 Aug 2;8:1342. doi: 10.3389/fpls.2017.01342 (PMC5539243; doi:10.3389/fpls.2017.01342)
Supplement: Supplementary file 2 [file Table_2.DOCX]

Supplementary Material

**Identification and characterization of (3*Z*):(2*E*)-hexenal isomerases from cucumber**

Eleni A. Spyropoulou, Henk L. Dekker, Luuk Steemers, Jan H. van Maarseveen, Chris G. de Koster, Michel A. Haring Robert C. Schuurink, Silke Allmann*,

* Correspondence: Corresponding Author: [S.Allmann@uva.nl](mailto:S.Allmann@uva.nl)

# Supplementary Figures and Tables

**Table S1:** List of identified proteins in active and non-active trypsin digested gel slices after initial purification (partI). Separate Excel file

**Table S2**: List of identified proteins in active and non-active fraction after final purification (partII). HI Cs033090 is marked in green. Common contaminants were removed from this list.

**Table S3**: Primer sequences used for cloning of putative hexenal isomerases

**Table S4:** Primer sequences used for qPCR

**Figure S1**: Partial purification of (3Z):(2E)-enal isomerase activity from peeled cucumber fruits (Part I).

**Figure S2**: Partial purification of (3Z):(2E)-enal isomerase activity from peeled cucumber fruits (Part II).

**Figure S3**: Results of the partial purification (part I).

**Figure S4**: Protein sequence of the HI (Cs033090) identified from the final purification of Cucumber fruits (part II; Figure S2).

**Figure S5**: Brilliant Blue G Colloidal stained SDS-PAGE of BSA standards and purified recombinant protein of HI homologs from cucumber.

## Supplementary Tables

**Table S2**: List of identified proteins in active and non-active fraction after final purification (partII). HI Cs033090 is marked in green. Common contaminants were removed from this list.

| Protein accession | Protein description | Protein mass | ***Active Fraction*** | | | | ***Non-active Fraction*** | | | |
| --- | --- | --- | --- | --- | --- | --- | --- | --- | --- | --- |
|  |  |  | *Run I* | | *Run II* | | *Run I* | | *Run II* | |
|  |  |  | Protein score | # peptides | Protein score | # peptides | Protein score | # peptides | Protein score | # peptides |
| Cucsa.362680.1 | arginase | 37147 | 631 | 25 | 1354 | 37 | 292 | 10 | 165 | 6 |
| Cucsa.362680.2 | arginase | 37147 | 631 | 25 | 1354 | 37 | 292 | 10 | 165 | 6 |
| Cucsa.362680.3 | arginase | 37147 | 631 | 25 | 1354 | 37 | 292 | 10 | 165 | 6 |
| Cucsa.188190.1 | PDI-like 1-1 | 57274 | 428 | 15 | 82 | 6 | 79 | 6 | 36 | 2 |
| Cucsa.033090.1 | RmlC-like cupins superfamily protein | 37431 | 373 | 12 | 1302 | 31 | 90 | 2 | 35 | 1 |
| Cucsa.160160.1 | general regulatory factor 2 | 29644 | 332 | 11 | 149 | 3 | 98 | 2 |  |  |
| Cucsa.044790.1 | fumarylacetoacetase, putative | 47598 | 166 | 10 | 69 | 4 | 188 | 10 | 97 | 5 |
| Cucsa.044790.2 | fumarylacetoacetase, putative | 36190 | 166 | 10 | 69 | 4 | 188 | 10 | 97 | 5 |
| Cucsa.166960.1 | histidinol dehydrogenase | 53521 | 259 | 10 | 94 | 4 | 117 | 4 | 101 | 2 |
| Cucsa.166960.2 | histidinol dehydrogenase | 53521 | 259 | 10 | 94 | 4 | 117 | 4 | 101 | 2 |
| Cucsa.166960.3 | histidinol dehydrogenase | 53521 | 259 | 10 | 94 | 4 | 117 | 4 | 101 | 2 |
| Cucsa.166960.4 | histidinol dehydrogenase | 53521 | 259 | 10 | 94 | 4 | 117 | 4 | 101 | 2 |
| Cucsa.166960.5 | histidinol dehydrogenase | 47438 | 259 | 10 | 94 | 4 | 117 | 4 | 101 | 2 |
| Cucsa.319770.1 | nitrilase-like protein 1 | 33565 | 103 | 10 | 69 | 2 |  |  |  |  |
| Cucsa.166800.1 | damaged DNA binding protein 1A | 123254 | 144 | 10 |  |  | 135 | 4 |  |  |
| Cucsa.311590.1 | general regulatory factor 2 | 29222 | 312 | 10 |  |  |  |  |  |  |
| Cucsa.313250.1 | Peptidase M20/M25/M40 family protein | 47804 | 428 | 9 | 139 | 6 |  |  |  |  |
| Cucsa.132900.12 | threonine aldolase 1 | 40664 | 275 | 9 | 112 | 3 |  |  |  |  |
| Cucsa.132900.2 | threonine aldolase 1 | 38852 | 275 | 9 | 112 | 3 |  |  |  |  |
| Cucsa.132900.7 | threonine aldolase 1 | 38084 | 275 | 9 | 112 | 3 |  |  |  |  |
| Cucsa.132900.10 | threonine aldolase 1 | 33376 | 275 | 9 |  |  |  |  |  |  |
| Cucsa.132900.12 | threonine aldolase 1 | 31551 | 275 | 9 |  |  |  |  |  |  |
| Cucsa.303280.1 | Peptidase M1 family protein | 103089 | 213 | 8 | 51 | 3 | 194 | 11 | 63 | 2 |
| Cucsa.097300.1 | Class I glutamine amidotransferase-like superfamily protein | 42399 | 343 | 8 | 73 | 2 |  |  |  |  |
| Cucsa.197230.1 | aldehyde dehydrogenase 10A8 | 55415 | 156 | 8 | 34 | 2 |  |  |  |  |
| Cucsa.197230.2 | aldehyde dehydrogenase 10A8 | 52784 | 156 | 8 | 34 | 2 |  |  |  |  |
| Cucsa.160760.1 | phosphoglucose isomerase 1 | 68439 | 107 | 8 | 73 | 1 | 1112 | 39 | 740 | 25 |
| Cucsa.273130.1 | general regulatory factor 7 | 29684 | 151 | 6 | 112 | 3 |  |  |  |  |
| Cucsa.273130.2 | general regulatory factor 7 | 29684 | 151 | 6 | 112 | 3 |  |  |  |  |
| Cucsa.273130.3 | general regulatory factor 7 | 29684 | 151 | 6 | 112 | 3 |  |  |  |  |
| Cucsa.147560.1 | general regulatory factor 9 | 29494 | 162 | 4 | 24 | 1 |  |  |  |  |
| Cucsa.395120.1 | glycine decarboxylase P-protein 1 | 114291 | 89 | 3 | 90 | 3 | 267 | 9 | 25 | 3 |
| Cucsa.091600.1 | mevalonate kinase | 41372 | 97 | 3 |  |  |  |  |  |  |
| Cucsa.160780.1 | general regulatory factor 8 | 28450 | 94 | 3 |  |  |  |  |  |  |
| Cucsa.160780.2 | general regulatory factor 8 | 27755 | 94 | 3 |  |  |  |  |  |  |
| Cucsa.160780.3 | general regulatory factor 8 | 26383 | 94 | 3 |  |  |  |  |  |  |
| Cucsa.218460.1 | aldehyde dehydrogenase 6B2 | 58209 | 72 | 2 | 36 | 1 | 91 | 4 |  |  |
| Cucsa.288710.2 | O-Glycosyl hydrolases family 17 protein | 46440 | 124 | 2 | 63 | 1 | 67 | 1 |  |  |
| Cucsa.288710.1 | O-Glycosyl hydrolases family 17 protein | 52600 | 93 | 2 | 63 | 1 | 67 | 1 |  |  |
| Cucsa.273340.1 | Zincin-like metalloproteases family protein | 90375 | 77 | 2 |  |  | 420 | 21 | 178 | 8 |
| Cucsa.139040.1 | alpha-glucan phosphorylase 2 | 96136 | 61 | 2 |  |  | 464 | 17 | 101 | 7 |
| Cucsa.139040.2 | alpha-glucan phosphorylase 2 | 96136 | 61 | 2 |  |  | 464 | 17 | 101 | 7 |
| Cucsa.139040.3 | alpha-glucan phosphorylase 2 | 96136 | 61 | 2 |  |  | 464 | 17 | 101 | 7 |
| Cucsa.139040.4 | alpha-glucan phosphorylase 2 | 96136 | 61 | 2 |  |  | 464 | 17 | 101 | 7 |
| Cucsa.254060.1 | pfkB-like carbohydrate kinase family protein | 41081 | 62 | 2 |  |  |  |  |  |  |
| Cucsa.254060.2 | pfkB-like carbohydrate kinase family protein | 39556 | 62 | 2 |  |  |  |  |  |  |
| Cucsa.254060.3 | pfkB-like carbohydrate kinase family protein | 39556 | 62 | 2 |  |  |  |  |  |  |
| Cucsa.368120.1 | ribulose-bisphosphate carboxylases | 31654 | 25 | 2 |  |  |  |  |  |  |
| Cucsa.111930.1 | manganese superoxide dismutase 1 | 26920 | 33 | 1 |  |  | 159 | 6 | 139 | 5 |
| Cucsa.089630.7 | xylose isomerase family protein | 53625 | 52 | 1 |  |  | 102 | 5 | 22 | 2 |
| Cucsa.089630.16 | xylose isomerase family protein | 53625 | 52 | 1 |  |  | 102 | 5 |  |  |
| Cucsa.194030.1 | S-adenosyl-L-homocysteine hydrolase | 53771 | 26 | 1 |  |  | 66 | 4 |  |  |
| Cucsa.256680.1 | S-adenosyl-L-homocysteine hydrolase | 54018 | 26 | 1 |  |  | 66 | 4 |  |  |
| Cucsa.255630.1 | cyanase | 18833 | 34 | 1 |  |  | 66 | 3 | 40 | 1 |
| Cucsa.089630.18 | xylose isomerase family protein | 53625 | 52 | 1 |  |  |  |  | 22 | 2 |
| Cucsa.249340.1 | purple acid phosphatase 26 | 55743 | 75 | 1 |  |  |  |  |  |  |
| Cucsa.392510.1 | ribulose-bisphosphate carboxylases | 13700 | 64 | 1 |  |  |  |  |  |  |
| Cucsa.089630.12 | xylose isomerase family protein | 53625 | 52 | 1 |  |  |  |  |  |  |
| Cucsa.089630.16 | xylose isomerase family protein | 52730 | 52 | 1 |  |  |  |  |  |  |
| Cucsa.089630.18 | xylose isomerase family protein | 44875 | 52 | 1 |  |  |  |  |  |  |
| Cucsa.135160.1 | mitochondrial lipoamide dehydrogenase 1 | 54549 | 49 | 1 |  |  |  |  |  |  |
| Cucsa.252370.1 | mitochondrial lipoamide dehydrogenase 1 | 53818 | 49 | 1 |  |  |  |  |  |  |
| Cucsa.089710.1 | FGGY family of carbohydrate kinase | 67275 | 36 | 1 |  |  |  |  |  |  |
| Cucsa.014060.1 | DNAse I-like superfamily protein | 45520 | 34 | 1 |  |  |  |  |  |  |
| Cucsa.111930.2 | manganese superoxide dismutase 1 | 24042 | 33 | 1 |  |  |  |  |  |  |
| Cucsa.395610.1 | no pollen germination related 1 | 81439 | 25 | 1 |  |  |  |  |  |  |
| Cucsa.395610.2 | no pollen germination related 1 | 81439 | 25 | 1 |  |  |  |  |  |  |
| Cucsa.395610.3 | no pollen germination related 1 | 67076 | 25 | 1 |  |  |  |  |  |  |
| Cucsa.288880.1 | NADP-malic enzyme 3 | 65436 | 23 | 1 |  |  |  |  |  |  |
| Cucsa.288880.2 | NADP-malic enzyme 3 | 55427 | 23 | 1 |  |  |  |  |  |  |
| Cucsa.091190.1 | Zinc finger, RING-type;Transcription factor jumonji/aspartyl beta-hydroxylase | 108030 | 19 | 1 |  |  |  |  |  |  |
| Cucsa.156410.1 | Rubisco methyltransferase family protein | 59137 |  |  | 22 | 2 |  |  |  |  |
| Cucsa.156410.2 | Rubisco methyltransferase family protein | 59137 |  |  | 22 | 2 |  |  |  |  |
| Cucsa.156410.3 | Rubisco methyltransferase family protein | 59137 |  |  | 22 | 2 |  |  |  |  |
| Cucsa.156410.4 | Rubisco methyltransferase family protein | 59137 |  |  | 22 | 2 |  |  |  |  |
| Cucsa.156410.5 | Rubisco methyltransferase family protein | 59137 |  |  | 22 | 2 |  |  |  |  |
| Cucsa.197230.3 | aldehyde dehydrogenase 10A8 | 50140 |  |  | 34 | 2 |  |  |  |  |
| Cucsa.197230.4 | aldehyde dehydrogenase 10A8 | 43301 |  |  | 34 | 2 |  |  |  |  |
| Cucsa.101050.1 | Ribosomal protein L17 family protein | 18261 |  |  | 19 | 1 |  |  |  |  |
| Cucsa.101050.2 | Ribosomal protein L17 family protein | 15294 |  |  | 19 | 1 |  |  |  |  |
| Cucsa.232220.1 | Sugar isomerase (SIS) family protein | 62877 |  |  |  |  | 232 | 9 | 83 | 2 |
| Cucsa.232220.2 | Sugar isomerase (SIS) family protein | 62253 |  |  |  |  | 232 | 9 | 83 | 2 |
| Cucsa.041520.1 | Amidase family protein | 68154 |  |  |  |  | 71 | 3 |  |  |
| Cucsa.041520.2 | Amidase family protein | 61426 |  |  |  |  | 71 | 3 |  |  |
| Cucsa.345790.1 | Zinc-binding dehydrogenase family protein | 38434 |  |  |  |  | 78 | 3 |  |  |
| Cucsa.139010.1 | proliferating cell nuclear antigen 2 | 29743 |  |  |  |  | 41 | 2 |  |  |
| Cucsa.357280.1 | adenylosuccinate synthase | 54040 |  |  |  |  | 39 | 2 |  |  |
| Cucsa.094800.1 | translation initiation factor 3 subunit H1 | 39125 |  |  |  |  | 25 | 1 |  |  |
| Cucsa.094800.3 | translation initiation factor 3 subunit H1 | 31356 |  |  |  |  | 25 | 1 |  |  |
| Cucsa.129380.1 | U-box domain-containing protein kinase family protein | 92465 |  |  |  |  | 15 | 1 |  |  |
| Cucsa.129380.2 | U-box domain-containing protein kinase family protein | 91353 |  |  |  |  | 15 | 1 |  |  |
| Cucsa.129380.3 | U-box domain-containing protein kinase family protein | 91059 |  |  |  |  | 15 | 1 |  |  |
| Cucsa.236460.1 | RING/U-box superfamily protein | 60475 |  |  |  |  | 15 | 1 |  |  |
| Cucsa.236460.2 | RING/U-box superfamily protein | 60475 |  |  |  |  | 15 | 1 |  |  |
| Cucsa.236460.3 | RING/U-box superfamily protein | 60475 |  |  |  |  | 15 | 1 |  |  |
| Cucsa.302240.1 | Copper amine oxidase family protein | 81772 |  |  |  |  | 36 | 1 |  |  |
| Cucsa.397160.1 | Metallopeptidase M24 family protein | 55355 |  |  |  |  | 52 | 1 |  |  |
| Cucsa.397160.2 | Metallopeptidase M24 family protein | 52092 |  |  |  |  | 52 | 1 |  |  |
| Cucsa.100840.1 | histone deacetylase 14 | 35606 |  |  |  |  |  |  | 31 | 1 |
| Cucsa.321670.1 | cytochrome P450, family 88, subfamily A, polypeptide 3 | 56497 |  |  |  |  |  |  | 26 | 1 |
| Cucsa.339890.1 | polyribonucleotide nucleotidyltransferase, putative | 113744 |  |  |  |  |  |  | 20 | 1 |
| Cucsa.339890.2 | polyribonucleotide nucleotidyltransferase, putative | 95220 |  |  |  |  |  |  | 20 | 1 |
| Cucsa.339890.3 | polyribonucleotide nucleotidyltransferase, putative | 89801 |  |  |  |  |  |  | 20 | 1 |
| Cucsa.339890.4 | polyribonucleotide nucleotidyltransferase, putative | 40276 |  |  |  |  |  |  | 20 | 1 |

**Table S3**: Primer sequences used for cloning of putative hexenal isomerases

| **Gene** | **Accession number** | **Forward Primer (5’-3’)** | **Reverse Primer (5’-3’)** | **Resources for primers** |
| --- | --- | --- | --- | --- |
| Cs033080 | Cucsa.033080.1 | ATGGAGTTGAATTTGAAGCCAATGG | TCAACTATCTGAGGGAGGAACCAAGG | https://phytozome.jgi.doe.gov/pz/portal.html |
| Cs033090 | Cucsa.033090.1 | ATGGAGGCAATGAATCCCAAGCCTT | TTAAACCTTTGACCTAAAGAGCTTC | https://phytozome.jgi.doe.gov/pz/portal.html |
| Cs240840 | Cucsa.240840.1 | ATGGAAATCGATTTGACTCCTCAAC | CTAATTTGAAGGAGGGAAGAAGATC | https://phytozome.jgi.doe.gov/pz/portal.html |
| Cs078390 | Cucsa.078390.1 | ATGGAGGAGCAAAACTTGAAGGCAA | TTAATTTGTTATGTTTGACCTCAAA | https://phytozome.jgi.doe.gov/pz/portal.html |

**Table S4**: Primer sequences used for qPCR

| **Gene** | **Acession number ncbi** | **Forward Primer (5’-3’)** | **Reverse Primer (5’-3’)** | **Resources for primers** |
| --- | --- | --- | --- | --- |
| Cs033080 | XM_011652974 | GGAAGTGAAAGCGGGACAGT | CTAACAGAGGGTGTGTGGTGG | designed with primer-blast from ncbi |
| Cs033090 | XM_004151456.2 | TGGGTGAAACCAAAAGGGCT | GGGAGGGATTGGGATGGTTG | designed with primer-blast from ncbi |
| Cs240840 | XM_004150346.2 | GAGCACCGAAATGGAATGGC | CGGGAGGTTCTTCGTGTTCA | designed with primer-blast from ncbi |
| Cs078390 | XM_004139666.2 | TGGAGAAACTGGCTTGGCTG | TTGCTCGACGACGACAATCC | designed with primer-blast from ncbi |
| Actin-7like | XM_004173778.1. | GCTGGCATATGTTGCTCTTG | GAATCTCTCAGCTCCGATGG | Shi et *al*., 2015 |

## Supplementary Figures


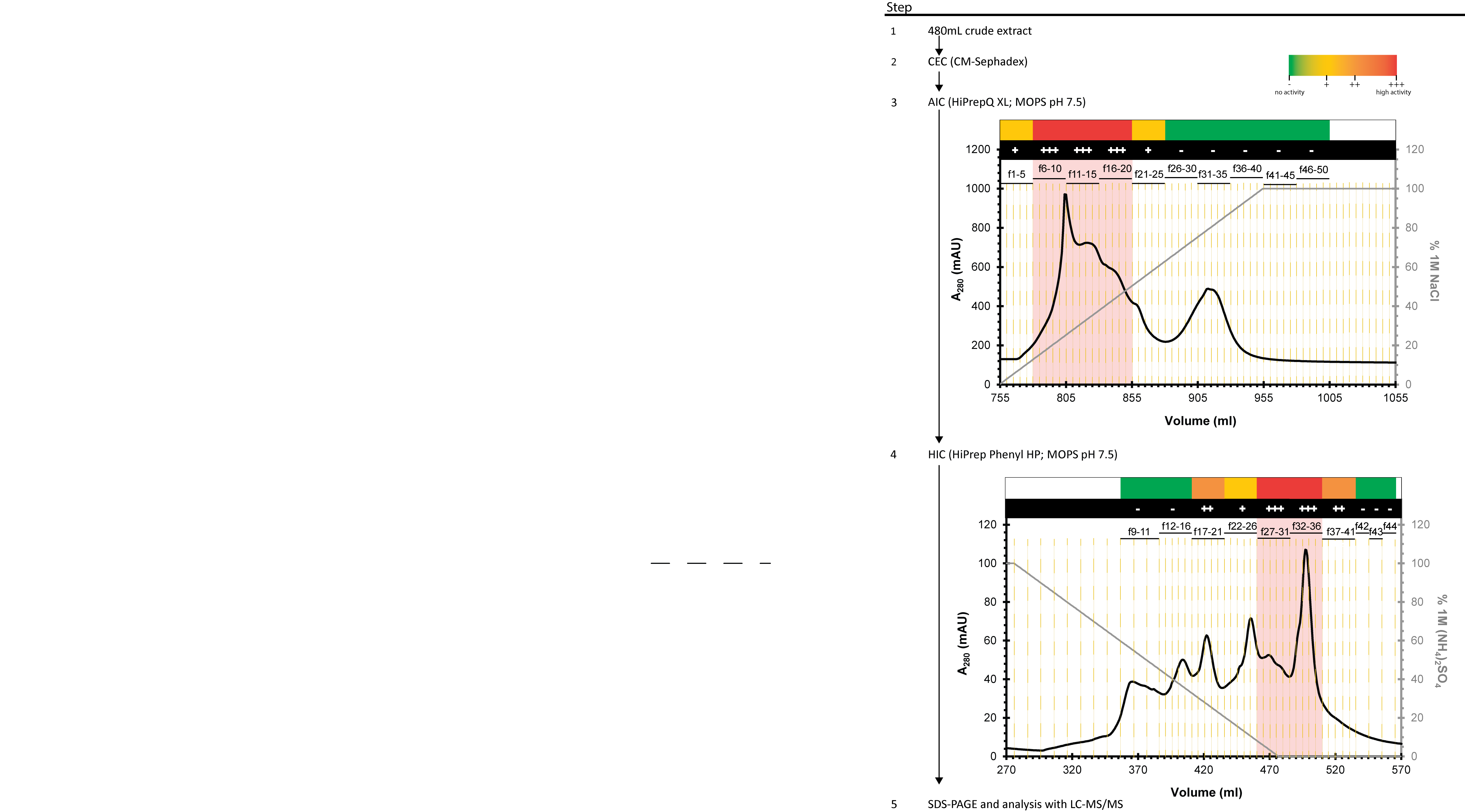


**Supplementary Figure 1.** **Partial purification of (3*Z*):(2*E*)-enal isomerase activity from peeled cucumber fruits (Part I).** Cucumber extract from 2 fruits was filtered and centrifuged (**Step 1**) and the crude supernatant was subjected to cation exchange chromatography (CEC) in a batch procedure (**Step 2**). Isomerase activity was recovered from the supernatant which was subsequently applied on a HiPrepQ XL column for anion exchange chromatography (AIC). Proteins were eluted with an increasing linear gradient of NaCl (0-1M) (**Step 3**) Combined active fractions were further separated by hydrophobic interaction chromatography (HIC) by loading them on a HiPrep Phenyl HP column in 1M (NH_4_)_2_SO_4_. Proteins were eluted with a linear gradient of decreasing (NH_4_)_2_SO_4_ (1-0M; **Step 4**). After precipitation proteins of the pooled active and a non-active fraction were separated by SDS-PAGE and analysed by LC-MS/MS (**Step 5**). Isomerase activity was tested by GC-ToF-MS either from a pool of fractions (indicated by a horizontal black line on top of the chromatogram) or from single fractions. Plus and minus symbols on top of the chromatogram as well as color code indicate the level of isomerase activity for single fractions or pooled fractions. Transparent red areas within the chromatogram mark those fractions that were used for further purification or for LC-MS-analysis. F, fractions; CEC, cation exchange chromatography; HIC, hydrophobic interaction chromatography.


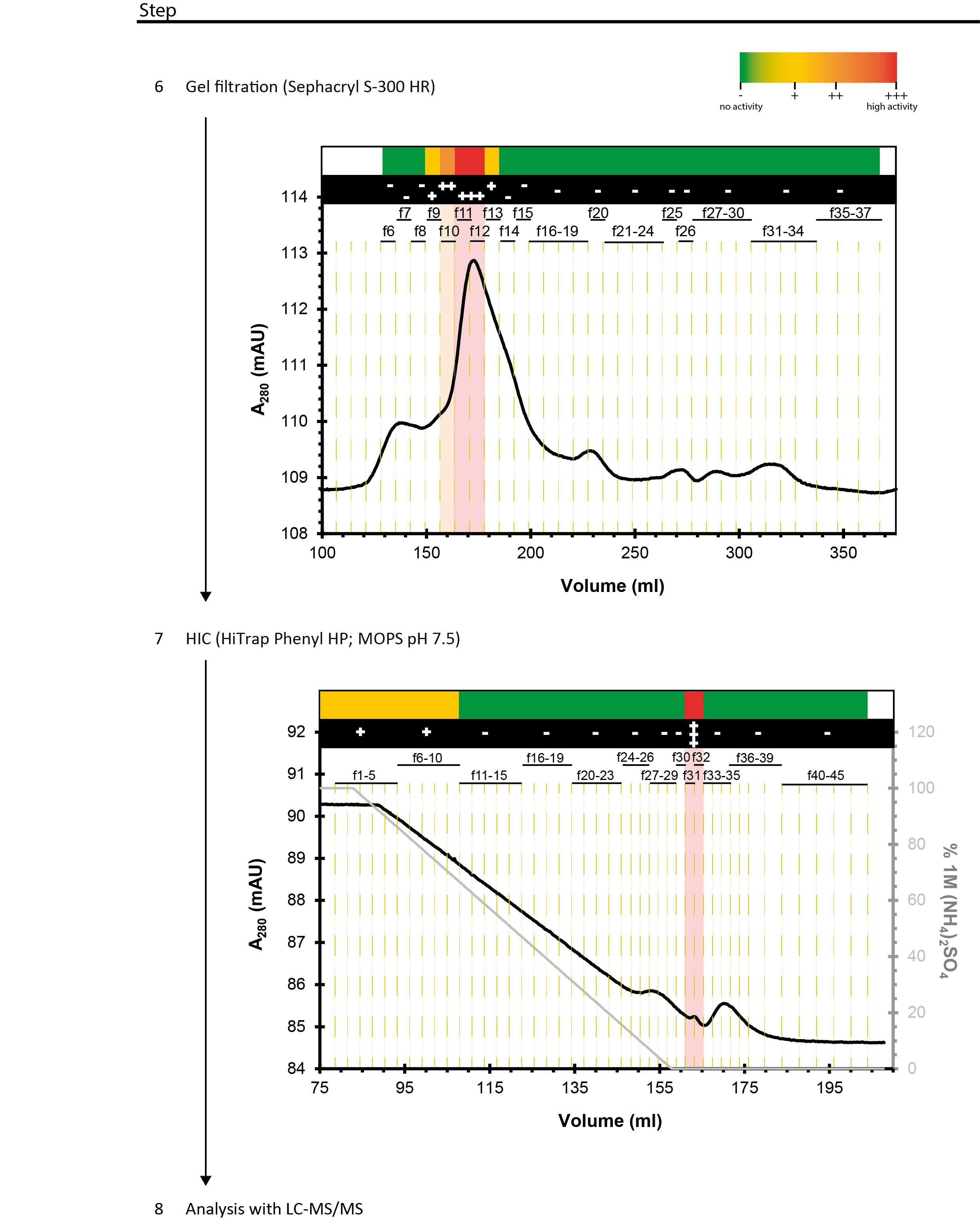


**Supplementary Figure 2. Partial purification of (3*Z*):(2*E*)-enal isomerase activity from peeled cucumber fruits (Part II).** 10mL of the pooled active fractions from step 4 was applied on a gel filtration column and proteins were eluted with a constant flow (**Step 6**). Combined active fractions were further separated by HIC on a HiTrap Phenyl HP column in 1M (NH_4_)_2_SO_4_. Proteins were eluted with a linear gradient of decreasing (NH_4_)_2_SO_4_ (1-0M; **Step 7**). After precipitation proteins of the pooled active and non-active fractions were analysed by LC-MS/MS (**Step 8**). Isomerase activity was tested by GC-ToF-MS either from a pool of fractions (indicated by a horizontal black line on top of the chromatogram) or from single fractions. Plus and minus symbols on top of the chromatogram as well as color code indicate the level of isomerase activity for single fractions or pooled fractions. Transparent red or orange areas within the chromatogram mark those fractions that were used for further purifications or for LC-MS-analysis. F, fractions; HIC, hydrophobic interaction chromatography.


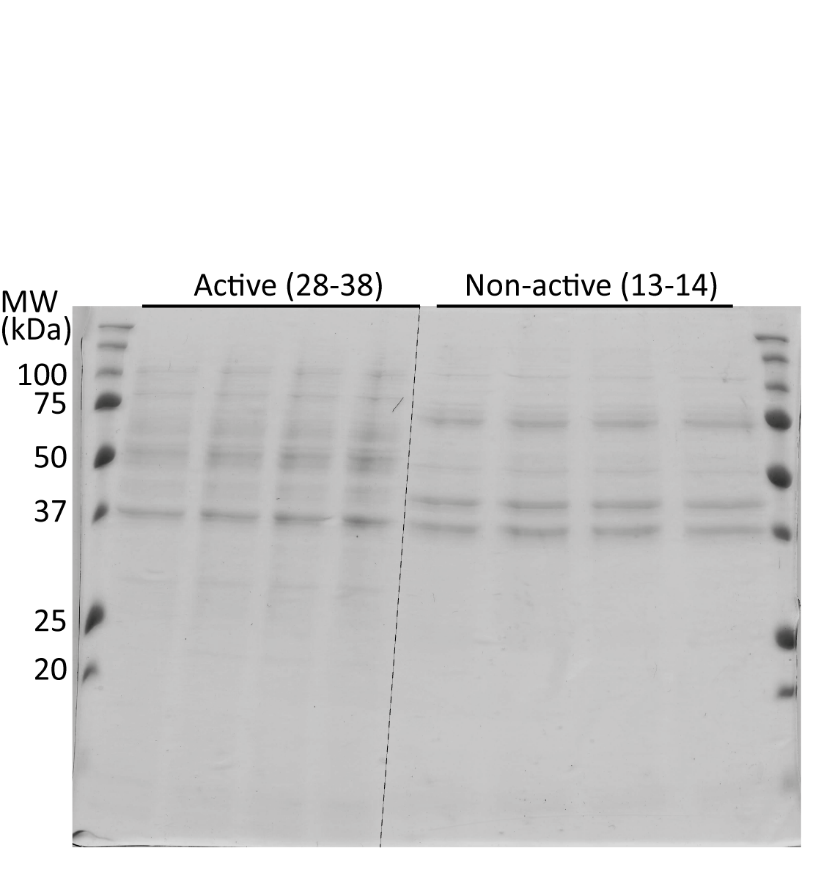


**Supplementary Figure 3. Results of the partial purification (part I).** Pooled active and non-active fractions were separated on SDS-PAGE and gel slices were in-gel digested for LC-MS analysis. For LC-MS a non-stained gel was used.


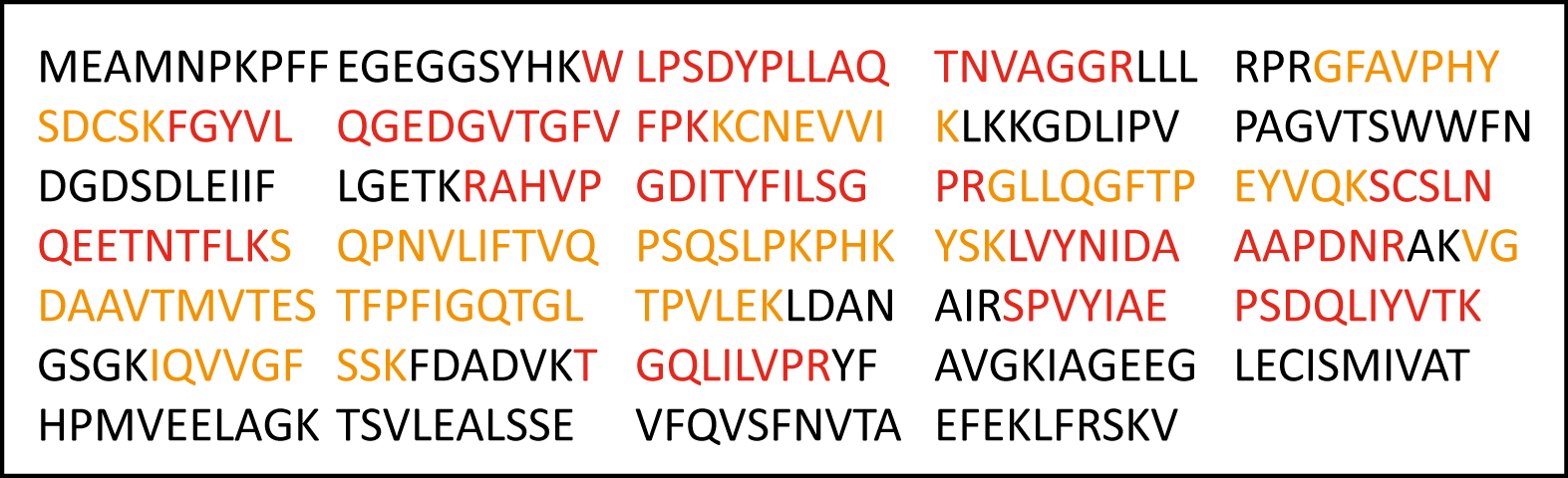


**Supplementary Figure 4. Protein sequence of the HI (Cs033090) identified from the final purification of Cucumber fruits (part II; Figure S2).** Peptides that were identified by LC-MS/MS are marked alternately in red and orange.


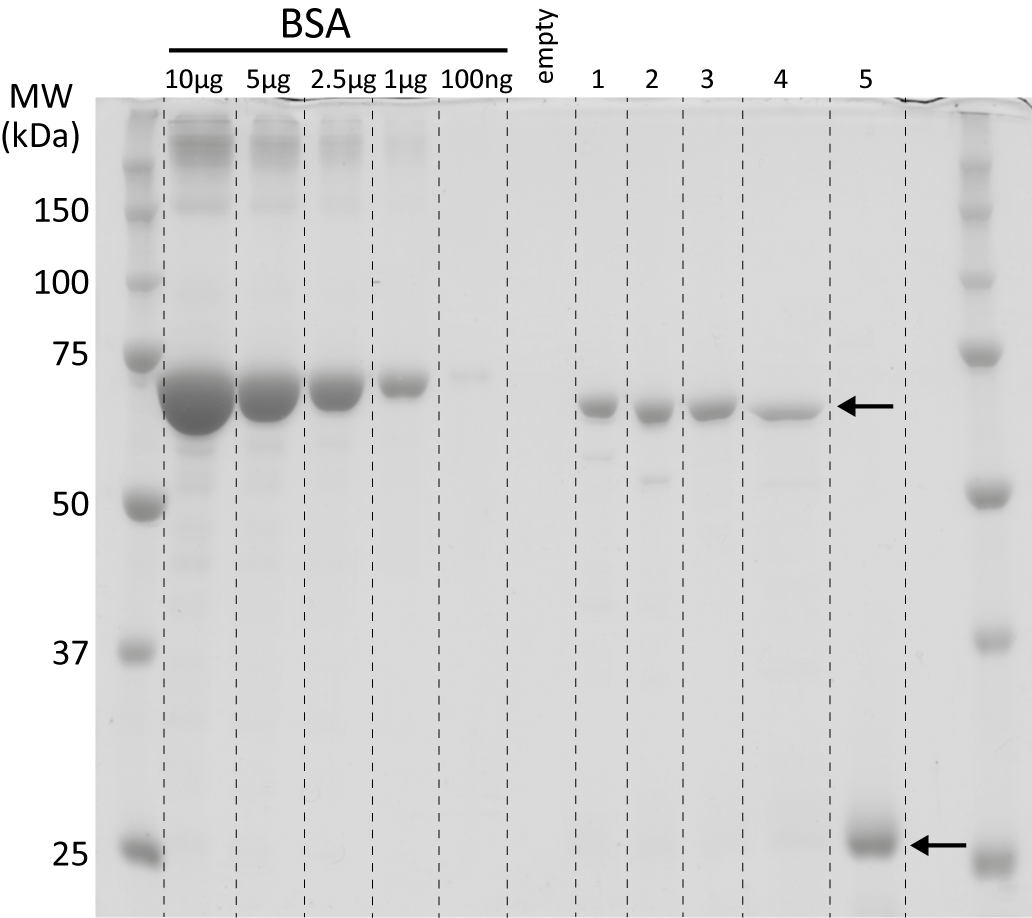


**Supplementary Figure 5. Brilliant Blue G Colloidal stained SDS-PAGE of BSA standards and purified recombinant protein of HI homologs from cucumber.** 2µL (1-3, 5) or 20µL (4) were loaded on the gel. Arrows mark bands at approx. 63 kDa (1-4; 37kDa HI + 26kDa GST); and at approx. 26kDa (5; 26kDa GST). 1, Cs033080; 2, Cs033090; 3, Cs240840; 4, 078390; 5, Empty vector containing GST.
